# Morphometric assessment of the left inferior phrenic vein in patients with portal hypertension

**DOI:** 10.1038/s41598-022-19610-w

**Published:** 2022-09-10

**Authors:** Yoshimi Fujii, Jun Koizumi, Yuka Sekiguchi, Shun Ono, Tatsuya Sekiguchi, Takuya Hara, Jun Hashimoto

**Affiliations:** 1 Department of Diagnostic Radiology, Fujisawa City Hospital, Fujisawa 2-6-1, Fujisawa-shi, Kanagawa 251-8550 Japan; 2 Department of Diagnostic Radiology and Radiation Oncology, School of Medicine, Chiba University, Chiba, Japan; 3 Department of Diagnostic Radiology, School of Medicine, Tokai University, Kanagawa, Japan

**Keywords:** Anatomy, Gastroenterology

## Abstract

The left inferior phrenic vein (LIPV) is a major drainage vessel of gastric varices and serves as an important conduit in endovascular treatment for gastric varices. The narrowing of LIPV has been empirically demonstrated and sometimes hinders catheter insertion for the treatment of gastric varices. We herein investigated the morphology of narrowed LIPV in patients with portal hypertension. Venograms of LIPV on 25 patients with gastric varices (15 males; 10 females; age range, 45–79 years with a mean of 67 years) were retrospectively reviewed, the following four parameters were measured: the diameter of LIPV, the diameter of narrowed LIPV, the narrowing rate, and the distance to narrowed LIPV from the left renal vein. On all 25 venograms, a narrowing was detected just above the common trunk with the left adrenal vein. The diameter of LIPV was 9.0 ± 4.2 mm, the diameter of narrowed LIPV was 5.1 ± 2.3 mm, the narrowing rate was 40.6 ± 16.0%, and the distance to narrowed LIPV from the left renal vein was 20.0 ± 7.4 mm. This anatomical information about the narrowing of LIPV may contribute to the safe and efficacious treatment of gastric varices.

## Introduction

Gastroesophageal varices are a common and life-threatening complication in patients with liver cirrhosis. Although gastric varices are less likely to bleed than esophageal varices, they are more severe when they bleed and, thus, have a higher mortality rate^1–3^. The majority of gastric varices are fed by the left gastric vein, posterior gastric vein, and short gastric vein, drain into the left inferior phrenic vein (LIPV), and form a portosystemic shunt^1,3–5^.

**Figure 1:**
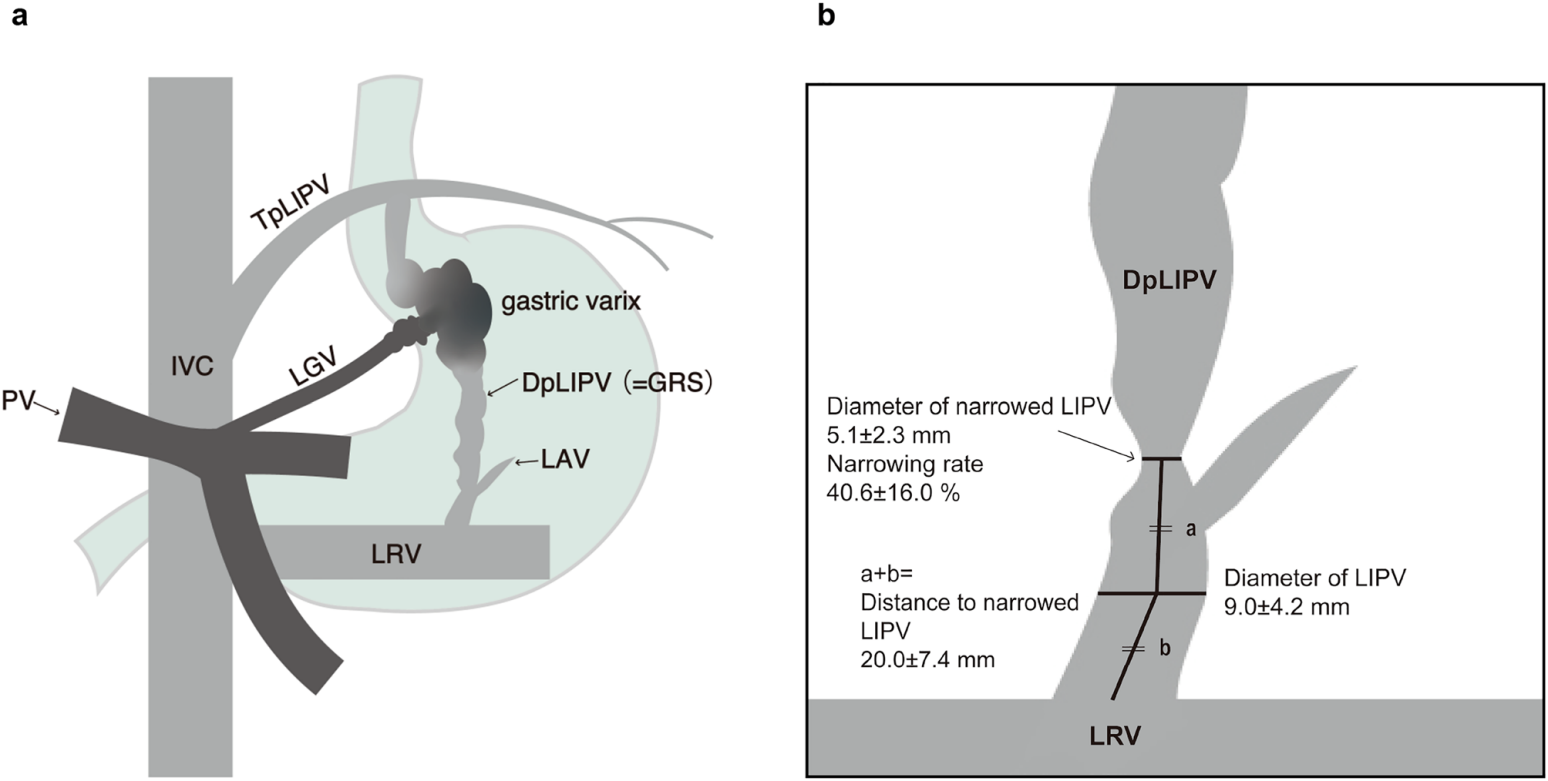
(**a**) Basic anatomy of LIPV and portosystemic venous circulation in a patient with portal hypertension. LIPV flows into two veins, namely, the transverse and descending portions of LIPV. The descending portion of LIPV merges with the left adrenal vein and flows into left renal vein, which is called the gastro-renal shunt. (**b**) The results of measurements on venograms of LIPV. IVC, Inferior vena cava; PV, Portal vein; LGV, Left gastric vein; TpLIPV, Transverse portion of the left inferior phrenic vein; DpLIPV, Descending portion of the left inferior phrenic vein; GRS, gastro-renal shunt; LAV, left adrenal vein; LRV, left renal vein.

LIPV originates superior to the diaphragm and commonly flows into two veins; the transverse portion flows directly into the subdiaphragmatic portion of the inferior vena cava (IVC), and the descending portion generally merges with the left adrenal vein and flows into the left renal vein. The latter is called a gastro-renal shunt (GRS) in patients with gastric varices and is one of the most important collaterals in portal hypertension^1,6,7^ (Fig. 1).

LIPV is also a key structure in the treatment of gastric varices. Balloon-occluded retrograde transvenous obliteration (BRTO) is widely applied as an effective endovascular treatment for gastric varices^3,4,8–11^. In BRTO, a balloon catheter is inserted retrograde into the GRS and a sclerosing agent is injected through the catheter into the varices under balloon occlusion. The catheterization of LIPV is the first step in BRTO; however, operators sometimes experience difficulties because of narrowing near the outlet of LIPV, which prevents catheter manipulation.

The narrowing of LIPV has been empirically demonstrated, and a previous study indicated that it was caused by venous valves^11,12^. However, the frequency, location, and rate of narrowing have not yet been investigated. We herein examined the morphology of narrowed LIPV in patients with portal hypertension using venograms during BRTO and investigated whether it correlates with the severity of portal hypertension. This information may contribute to the safe and effective treatment of gastric varices^10,11^.

## Methods

A retrospective review was performed of the medical records and images from 43 consecutive patients with gastric varices who underwent BRTO in our institution between July 2009 and July 2021.

In the BRTO procedure, a 5- or 6- French catheter was inserted through the right femoral vein or right internal jugular vein and introduced into LIPV via the left renal vein. To ensure proper catheterization and to examine the conformation of the GRS, venography was performed by manual injection with approximately 5 mL of iodinated contrast material at the outlet of LIPV.

Ten out of 43 patients were excluded from the present study because venography using carbon dioxide was performed before conventional venography using iodinated contrast material. Another eight patients were excluded because LIPV was not clearly visible on venograms. Therefore, we retrospectively reviewed venograms of LIPV from 25 patients.

### Measurements

**Figure 2:**
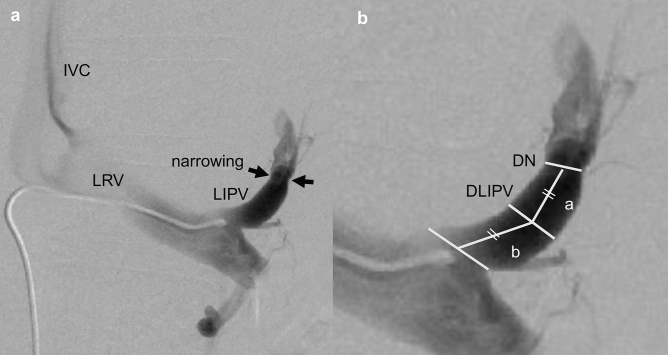
(**a**) Venogram of LIPV during BRTO. (**b**) The diameter of LIPV was measured at the midpoint between narrowing and the outlet of LIPV. The diameter of narrowed LIPV was measured at a narrow portion that was the closest to the outlet of LIPV. The distance to narrowed LIPV was defined as the distance from narrowed LIPV to the midpoint (= a) + the distance from the midpoint to the outlet of LIPV (= b). IVC, inferior vena cava; LRV, left renal vein; LIPV, left inferior phrenic vein; LARV, left adrenal vein; DN, Diameter of narrowed LIPV; DLIPV, Diameter of the left inferior phrenic vein.

We confirmed the depiction of LIPV, anatomical variants, such as GRS drainages via gonadal vein or duplicated GRS, and the presence of narrowing^11^. We measured the parts, as shown in Fig. 2. Measurements were defined as follows:

- Diameter of LIPV (DLIPV) (mm)
  The diameter of the midpoint between the narrowing and outlet of LIPV, which was the largest part of the common trunk.
- Diameter of narrowed LIPV (DN) (mm)
  The diameter of the narrowest part that was the closest to the outlet of LIPV.
- The narrowing rate was calculated as (1-DN/DLIPV) $\times 100$ (%)
- Distance to narrowing (mm)
  The distance from a narrowing to the midpoint (= a) + distance from the midpoint to the superior border of the left renal vein (= b)

The diameter of LIPV, the diameter of narrowed LIPV and the narrowing rate were also measured on axial enhanced CT images that were taken before the procedure. Pre-procedural intravenous dynamic CT was performed (Sensation Cardiac, Siemens, Erlangen, Germany) with an intravenous bolus injection of 100 mL of 350 mg I/mL iodinated contrast material at a flow rate of 3 mL/s. The portal venous phase axial CT images were used for measurements. The diameter of LIPV was measured at the largest part of the common trunk and the diameter of narrowed LIPV was measured at the narrowest part that was the closest to the outlet of LIPV.

The relationship between measurement variables and Child–Pugh score, diameter of portal vein, total bilirubin level, and splenic volume were examined^13–15^. Automatic splenic volumetry was performed using enhanced CT images with dedicated software (ZIO Station System, Amin).

### Statistical analysis

Statistical analyses were performed using Modified R Commander (Ver 4.2.1). Normality was examined using Shapiro–Wilk normality test and homogeneity of variance was examined using Levene's test. Four measurement variables (the diameter of LIPV, the diameter of narrowed LIPV, the narrowing rate, and the distance to narrowing from the left renal vein) were compared between male and female patients using two sample *t*-test, and Welch two sample *t*-test, and Wilcoxon rank sum test depending on whether the distribution followed a normal pattern and homogeneity of variance.

Three measurement variables (the diameter of LIPV, the diameter of narrowed LIPV, and the narrowing rate) were examined for differences between on venograms and on axial enhanced CT images using paired *t*-test and Wilcoxon signed rank test depending on whether the distribution followed a normal pattern. The relationships between four measurement variables and Child–Pugh score, diameter of portal vein, total bilirubin level and spleen volumes were examined using Pearson’s product-moment correlation and Spearman’s rank correlation depending on whether the distribution followed a normal pattern. In all analyses, p values $<$ 0.05 were considered to be significant.

### Ethics declarations

The present study was approved by the ethical review board of Tokai University Hospital (No.21-104) and conducted in accordance with the “Ethical Principles for Medical Research Involving Human Subjects” of the Ministry of Health, Labor and Welfare and the Ministry of Education, Culture, Sports, Science and Technology of Japan. Due to the retrospective nature of the study, the requirement for written informed consent was waived by the ethical review board of Tokay University Hospital and study information and the contact address were published on the website of the hospital.

## Results

Among the 25 patients examined, 15 were males and 10 were females. Patients ranged in age between 45 and 79 years, with a mean of 67 years. No significant differences were observed in age between males and females (*p* = 0.34). Child–Pugh score was significantly higher in males (*p* = 0.0061). (Supplementary Table S1) In BRTO, catheterization into LIPV was successful in all cases, and venograms well depicted the lower part of LIPV. The left adrenal vein was delineated in five cases. No anatomical variants were observed on venograms. On all 25 venograms, a narrowing was observed just above the common trunk.

### Measurements (Supplementary Table S1)

**Figure 3:**
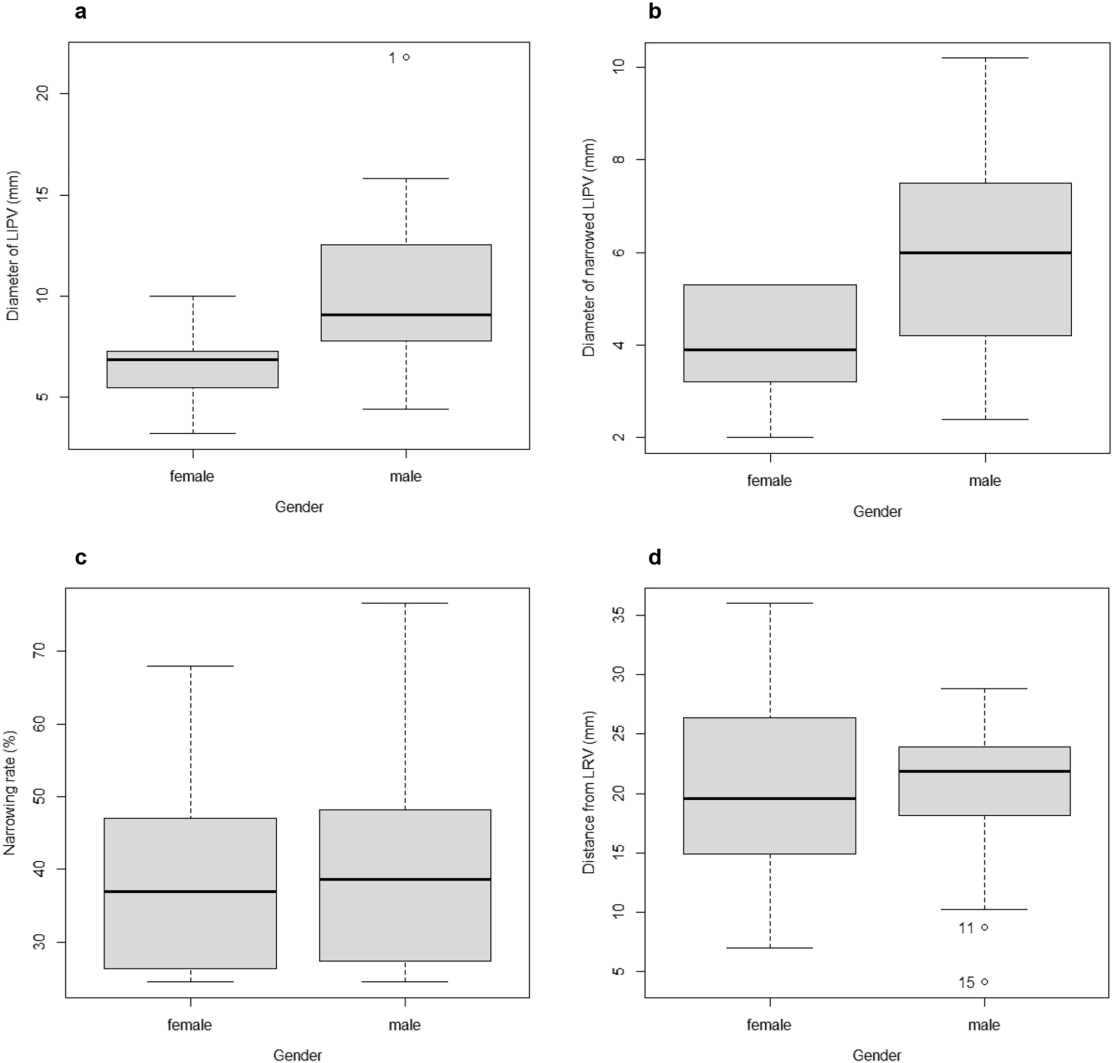
(**a**) Box plot of the diameter of LIPV in relation to sex (*p* = 0.0056). (**b**) Box plot of the diameter of narrowed LIPV in relation to sex (*p* = 0.013). c Box plot of the narrowing rate in relation to sex (*p* = 0.55). d Box plot of the distance from LRV in relation to sex (*p* = 0.83). The diameter of LIPV and the diameter of narrowed LIPV were significantly larger in males than in females, whereas no significant differences were observed in the narrowing rate or distance to narrowing from the left renal vein.

The diameter of LIPV ranged between 3.2 and 21.8 mm (4.4–21.8 for males and 3.2–10.0 for females), with a mean of 9.0 mm (10.6 for males and 6.6 for females) and standard deviation of 4.2 mm (4.5 for males and 1.9 for females) A significant difference was observed in the diameter of LIPV between males and females (*p* $$=0.0056)$$. with a 95% confidence interval of − 6.75 to − 1.32 mm (Fig. 3a).

The diameter of narrowed LIPV ranged between 2.0 and 10.2 mm (2.4–10.2 for males and 2.0–5.3 for females), with a mean of 5.1 mm (5.9 for males and 3.9 for females) and standard deviation of 2.3 mm (2.5 for males and 1.2 for females). A significant difference was observed in the diameter of narrowed LIPV between males and females (*p* $$=0.013)$$. with a 95% confidence interval of − 3.56 to − 0.48 mm (Fig. 3b).

The narrowing rate ranged between 24.5 and 76.6% (24.6–76.6 for males and 24.5–67.9 for females), with a mean of 40.6% (41.7 for males and 38.9 for females) and standard deviation of 16.0% (17.3 for males and 14.5 for females). A significant difference was not observed in the narrowing rate between males and females (*p* $$=0.55)$$ (Fig. 3c).

The distance to narrowing from the left renal vein ranged between 4.1 and 36.0 mm (4.1–28.8 for males and 7.0–36.0 for females), with a mean of 20.0 mm (19.8 for males and 20.3 for females) and standard deviation of 7.4 mm (7.2 for males and 8.2 for females). A significant difference was not observed in the distance to narrowing between males and females (*p* $$=0.83)$$ (Fig. 3d).

There was no significant difference in the diameter of LIPV (*p* = 0.66) or the narrowing rate (*p* = 0.074) between on venograms and on axial CT images. There was a significant difference in the diameter of narrowed LIPV (*p* = 0.029) with a 95% confidence interval of − 1.65 to 0.098 mm.

**Figure 4:**
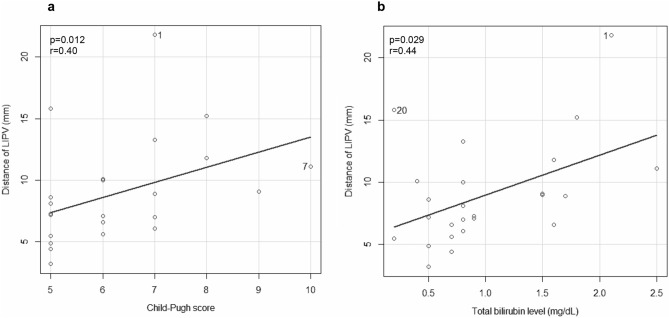
(**a**) Correlation between Child–Pugh score and the diameter of LIPV. The diameter of LIPV was significantly correlated (*p* = 0.012) with Child-Pugh score. The correlation coefficient was r $=$ 0.40. (**b**) Correlation between total bilirubin level and the diameter of LIPV. The diameter of LIPV was significantly correlated (*p* = 0.029) with total bilirubin levels. The correlation coefficient was r $=$ 0.44.

Among measurement variables, the diameter of LIPV and Child–Pugh score (*p* = 0.012) and the diameter of LIPV and total bilirubin level (*p* = 0.029) and were significantly correlated (Fig. 4). The correlation coefficients were r = 0.40 and r = 0.44, respectively. No other correlations were found between measurement variables and portal vein diameter, spleen volumes, total bilirubin level, or Child–Pugh score.

## Discussion

We examined venograms during BRTO for morphometric assessment of LIPV in patients with portal hypertension. All venograms of LIPV showed a narrowing just above the common trunk. The diameter of LIPV and narrowed LIPV were significantly larger in males than in females, whereas no significant differences were observed in the narrowing rate or the distance to narrowed LIPV from the left renal vein.

LIPV is rarely discussed in classical anatomy textbooks^16^. However, in recent years, with the development of endoscopic and endovascular treatments for esophageal and gastric varices, LIPV, which serves as an important portosystemic shunt in patients with portal hypertension, has been attracting increasing attention^12,17–19^. Loukas et al. classified and described variations in the origins and distributions of LIPV in cadavers^19^. Among 300 cadaveric specimens, 37% of LIPV drained into the IVC below the diaphragm, 25% into the left suprarenal vein, 15% into the left renal vein, 14% into the left hepatic vein, and 1% into both the IVC and left suprarenal vein. On the other hand, Araki et al. reviewed venograms obtained during adrenal vein sampling and reported that LIPV merged with the left adrenal vein in 92.8% of cases, and the mean distance from the confluence to the renal vein was 16.4 mm, with a standard deviation of 4.7 mm^12^. They also noted that 87.2% of patients without portal hypertension had narrowed LIPV, which was presumably formed by the valves. Saad et al. reviewed the BRTO technique and reported a web-like narrowing at the junction of LIPV with the common trunk, which made catheter insertion difficult^11^.

The present study is the first to describe morphometric features of LIPV and its narrowing. Since LIPV serves as a major portosystemic shunt, it was assumed that the diameter of LIPV correlates with the severity of portal hypertension. However, only total bilirubin level and Child–Pugh score correlated with the diameter of LIPV in the present study. Other factors should be considered to determine the association between the diameter of the LIPV and portal hypertension, including portal blood flow, portal pressure, and the degree of development of collateral pathways.

The results revealed the presence of a narrowing just above the common trunk of LIPV. There were no significant differences in the diameter of LIPV or narrowing rate between on venograms and on axial CT images. On the other hand, the diameter of narrowed LIPV was significantly larger on CT images (6.1 ± 2.3 mm) than on venograms (5.1 ± 2.3 mm) (*p* = 0.03). However, considering that the 95% confidence interval was − 1.65 to 0.098 mm, the difference of the diameter of narrowed LIPV between on venograms and on CT images was not considered clinically significant.

The mean distance to narrowed LIPV from the left renal vein was 20.0 mm with a standard deviation of 7.4 mm. This was longer than that previously reported by Araki et al.^12^, and may be attributed to differences in patient characteristics. The study by Araki et al. examined patients without portal hypertension, whereas we investigated and obtained measurements from patients with portal hypertension. LIPV may be dilated and tortuous in patients with portal hypertension, and, thus, the distance to narrowed LIPV from the left renal vein may be longer.

Based on the results obtained, In BRTO, careful catheter manipulation is required to pass through a narrowing of LIPV that is located at approximately 2 cm from the left renal vein. Once a catheter passes, the narrowing makes a very effective choke point^11^. In BRTO, it is essential for successful treatment that GRS is completely balloon occluded before injecting sclerosant into gastric varices. A balloon placed just above the narrowing can be stable and adequately occlude GRS^10,11^.

The present study has some limitations. First, the sample size was small. Second, venograms of LIPV during BRTO were obtained following a manual contrast injection in order to prevent rupture of the vein. Differences in the volume of contrast material and the injection speed may affect the depiction of LIPV and its narrowing. Moreover, we speculated that the narrowing of LIPV was formed by venous valves (downward-flow valves), which was consistent with the reason for the difficulty of catheter passage over the narrowing. However, the presence of venous valves has not yet to be confirmed histologically; therefore, further investigations on cadavers are needed.

## Conclusion

Anatomical information on LIPV and its narrowing may contribute to safe and efficacious treatment for gastric varices. The present results revealed the presence of narrowing just above the common trunk of LIPV in patients with portal hypertension. The mean distance to narrowed LIPV from the left renal vein was approximately 2 cm. Careful catheter manipulation is required in this area.

## Supplementary Information

Supplementary Information.

## Data Availability

The anonymized datasets are provided upon reasonable request from the corresponding author.

## References

[1] Watanabe K, Kimura K, Matsutani S, Ohto M, Okuda K (1988). Portal hemodynamics in patients with gastric varices. Gastroenterology.

[2] Sarin SK, Lahori D, Saxena SP, Murthy NS, Makwana UK (1992). Prevalence, classification, and natural history of gastric varices: A long-term follow up study in 568 portal hypertension patients. Hepatology.

[3] Wani ZA, Bhat RA, Bhadoria AS, Maiwall R, Choudhury A (2015). Gastric varices: Classification, endoscopic and ultrasonographic management. J. Res. Med. Sci..

[4] Lipnik AJ, Pandhi MB, Khabbaz RC, Gaba RC (2018). Endovascular treatment for variceal hemorrhage: TIPS, BRTO, and combined approaches. Semin. Intervent. Radiol..

[5] Kiyosue H (2013). Multidetector CT anatomy of drainage routes of gastric varices: A pictorial review. Radiographics.

[6] Sharma M, Rameshbabu CS (2012). Collateral pathway in portal hypertension. J. Clin. Exp. Hepatol..

[7] Gaba RC, Couture PM, Lakhoo J (2015). Gastroesophageal variceal filling and drainage pathways: An angiographic description of afferent and efferent venous anatomic patterns. J. Clin. Imaging Sci..

[8] Kanagawa H (1996). Treatment of gastric fundal varices by balloon-occluded retrograde transvenous obliteration. J. Gastroenterol. Hepatol..

[9] Hirota S, Matsumoto S, Tomita M, Sako M, Kono M (1999). Retrograde transvenous obliteration of gastric varices. Radiology.

[10] Saad WEA (2012). Balloon-occluded retrograde transvenous obliteration of gastric varices: Concept, basic techniques, and outcomes. Semin. Intercent. Radiol..

[11] Saad WEA, Kitanosono T, Koizumi J, Hirota S (2013). The conventional balloon-occluded retrograde transvenous obliteration procedure: Indications, contraindications, and technical applications. Tech. Vasc. Interventional Rad..

[12] Araki T, Imaizumi A, Okada H, Sasaki Y, Onishi H (2021). Anatomy of left inferior phrenic vein in patients without portal hypertension. Am. J. Roentgenol..

[13] Pareja QJS, Restrepo GJC (2016). Diagnostic methods in portal hypertension. Rev. Col. Gastroenterol..

[14] Gyoten K (2016). A novel predictor of posttransplant portal hypertension in adult-to-adult living donor liver transplantation: Increased estimated spleen/graft volume ratio. Transplantation.

[15] Hayashi H (2012). Combined measurements of serum bile acid level and splenic volume may be useful to noninvasively assess portal venous pressure. J. Gastroenterol..

[16] Standring S (2008). Gray’s Anatomy: The Anatomical Basis of Clinical Practice 40th Edition.

[17] Bonnette P, Hannoun L, Menegaux F, Calmat A, Cabrol C (1983). Anatomic study of the left inferior diaphragmatic vein (vena phrenica inferior sinistra). Bull. Assoc. Anat..

[18] Loukas M (2005). An anatomical classification of the variations of the inferior phrenic vein. Sur. Radiol. Anat..

[19] Lukas M (2008). A review of the distribution of the arterial and venous vasculature of the diaphragm and its clinical relevance. Folia Morphol..

