# Supplementary material for: Morphometric assessment of the left inferior phrenic vein in patients with portal hypertension
Source: Sci Rep. 2022 Sep 10;12:15275. doi: 10.1038/s41598-022-19610-w (PMC9464207; doi:10.1038/s41598-022-19610-w)
Supplement: Supplementary file 1 — Supplementary Information. [file 41598_2022_19610_MOESM1_ESM.pdf]

Morphometric assessment of the left inferior phrenic vein in patients with portal hypertension

Yoshimi Fujii*[1] • Jun Koizumi[2] • Yuka Sekiguchi[3] • Shun Ono[4] • Tatsuya Sekiguchi[5] • Takuya Hara[6] • Jun Hashimoto[7]

Corresponding author, Yoshimi Fujii*[1]

*1. Department of Diagnostic Radiology, Fujisawa City Hospital, Fujisawa 2-6-1, Fujisawa-shi, Kanagawa 251-8550, Japan. email: fujiiyosimi@gmail.com

2. Department of Diagnostic Radiology and Radiation Oncology, School of Medicine, Chiba University, Chiba, Japan. e-mail: koizumij7777777@gmail.com

3. Department of Diagnostic Radiology, School of Medicine, Tokai University, Kanagawa, Japan. email: firebyrdinscuderia@gmail.com

4. Department of Diagnostic Radiology, School of Medicine, Tokai University, Kanagawa, Japan. email: onsn25@outlook.jp

5. Department of Diagnostic Radiology, School of Medicine, Tokai University, Kanagawa, Japan.email: jpyqw100@gmail.com

6. Department of Diagnostic Radiology, School of Medicine, Tokai University, Kanagawa, Japan.email: takuya-_-ayukat@live.jp

7. Department of Diagnostic Radiology, School of Medicine, Tokai University, Kanagawa, Japan.email: junhashi@tokai-u.jp

Table S1. Baseline characteristics

	male (n=15)	female (n=10)
Mean age	68.5±7.9	64.9±10.46
BMI (kg/m^2)	23.6±4.5	23.2±3.8
Child-Pugh score A	5	10
score B	8	-
score C	1	-
NA	1	-

BMI, Body-mass index

Table S2. Results of measurements

	mean±SD	range
Diameter of LIPV on venograms (mm)	9.0±4.2	3.2-21.8
on CT images (mm)	9.3±3.3	4.6-17.8
Diameter of narrowed LIPV on venograms (mm)	5.1±2.3	2.0-10.2
on CT images (mm)	6.1±2.3	2.6-10.6
Narrowing rate on venograms (%)	40.6±15.1	24.4-76.6
on CT images (%)	34.4±10.9	12.4-50.9
Distance to narrowed LIPV from LRV (mm)	20.0±7.4	4.1-36.0

LIPV, left inferior phrenic vein; LRV, left renal vein
